# Drinking Water: NAS Reports on Perchlorate Safety

**DOI:** 10.1289/ehp.113-1257676

**Published:** 2005-07

**Authors:** Richard Dahl

A National Academy of Sciences (NAS) panel has issued a final report on the health implications of perchlorate ingestion, recommending a reference dose of 0.0007 milligrams per kilogram (mg/kg) body weight. But the debate over the health risks posed by the chemical, used by the Department of Defense as a rocket fuel additive, is far from over.

Perchlorate compounds have been used since the early 1900s, and environmental perchlorate contamination was first seen in 1985 in wells at California Superfund sites. Since then, perchlorate has been found in 35 states. In May 2004 the U.S. Environmental Protection Agency (EPA) estimated that more than 11 million Americans were drinking water from public supplies containing at least 4 parts per billion (ppb) perchlorate.

Scientists agree that perchlorate can interfere with the production of thyroid hormone since it competes for the uptake of iodide by the thyroid gland. But beliefs about what level of exposure constitutes a health risk vary widely. The Council on Water Quality (CWQ), a chemical and aerospace industry group, often cites a drinking water cutoff of 245 ppb. In contrast, California recommends that drinking water contain no more than 6 ppb perchlorate, and Massachusetts recommends that pregnant women and children not consume water with more than 1 ppb perchlorate.

The broad disagreement, coupled with the prospect of massive cleanup costs—estimated by some to be in the billions—prompted the government to ask the NAS for guidance. According to panel chair Richard B. Johnston, Jr., associate dean for research development at the University of Colorado School of Medicine in Denver, the 15-member group used as its starting point a September 2002 *EHP* study headed by Monte A. Greer of Oregon Health & Science University. This study was partially funded by the Perchlorate Study Group, an organization created by the Department of Defense and some of its contractors.

The Greer study concluded there was no inhibition of iodide uptake by the thyroid at 0.007 mg/kg body weight. The panel applied a 10-fold uncertainty factor to that figure to derive its own reference dose. “We took what we feel is the most conservative end point,” says Johnston. “It’s way short of any kind of harm.” Five weeks after the NAS made its report public, the EPA responded by adopting the NAS dose level and translating it into a drinking water equivalent level of 24 ppb.

But environmental groups have voiced heated disagreement with the NAS findings. Gina Solomon, a senior scientist at the Natural Resources Defense Council, says the report relied too heavily on a study she calls statistically flawed because of the small number of subjects (just 37). “As the effect [of perchlorate ingestion] gets more subtle, the size of the study group needs to be bigger to see if there’s an effect there or not,” she says.

Further, she says, the report suffers from tunnel vision: “[The NAS] should have been looking at the big picture on perchlorate, and they didn’t do that. The result was that their final report hinged entirely on one controversial industry study.”

Johnston responds that the panel also relied on four other clinical studies as well as several epidemiologic and perchlorate worker studies, all of which supported the Greer findings. And James Strock, a former secretary of the California Environmental Protection Agency who now works with the CWQ, says the NAS findings will provide state and federal regulators “a rare opportunity to promulgate regulations in a transparent manner, working simultaneously from information collected and considered by a world-class panel of experts.”

Johnston agrees, however, that more research will be helpful, especially on perchlorate’s effects on sensitive populations, such as pregnant women, nursing mothers, and infants. A study at Texas Tech University, published 1 April 2005 in *Environmental Science & Technology*, found that perchlorate levels in 36 samples of breast milk from nursing mothers in 18 states averaged 10.5 ppb, meaning the mothers were ingesting far more than 24 ppb. The study raises the possibility that some infants may be ingesting perchlorate at levels exceeding NAS and EPA safe doses.

Meanwhile, the controversy continues to play out, as described in an upcoming *EHP* commentary (doi:10.1289/ehp.8254, scheduled for publication in September 2005 and available in draft form at http://dx.doi.org/). Although the EPA has adopted the 24 ppb figure as an “official reference dose,” it’s not yet an enforceable standard, and Solomon says states are left to their own devices. “Some are following the EPA lead, and others are following the California lead,” she says. “This means that consumers in some states will be drinking water with higher levels of perchlorate than consumers in other states. And that’s unfortunate.”

